# Targeting Sleep to Improve Outcomes in Psychosis: Digital and Non-Pharmacological Interventions

**DOI:** 10.3390/medicina62071269

**Published:** 2026-06-30

**Authors:** Valentina Baldini, Martina Gnazzo, Giorgia Varallo, Diana De Ronchi, Lorenzo Pelizza, Marco Menchetti, Giuseppe Plazzi

**Affiliations:** 1 Department of Biomedical and Neuromotor Sciences, University of Bologna, 40126 Bologna, Italy; 2 Clinic of Child and Adolescent Neuropsychiatry, Department of Mental Health, Physical and Preventive Medicine, University of Campania “Luigi Vanvitelli”, 80138 Naples, Italy; 3 IRCCS Istituto Delle Scienze Neurologiche di Bologna (ISNB), 40139 Bologna, Italy; 4 Department of Biomedical, Metabolic and Neural Sciences, University of Modena and Reggio Emilia, 41125 Modena, Italy

**Keywords:** sleep, insomnia, psychosis, schizophrenia, first-episode psychosis, clinical high-risk

## Abstract

Sleep disturbances are among the most prevalent and clinically significant features observed across the psychosis spectrum, ranging from clinical high-risk (CHR) mental states to first-episode psychosis (FEP) and chronic schizophrenia. Far from being merely secondary phenomena, sleep difficulties—including insomnia, circadian rhythm disruption, altered sleep architecture, hypersomnia, and nightmare disorder—are increasingly acknowledged as transdiagnostic risk factors that may contribute to symptom severity, cognitive impairment, functional decline, and heightened suicidal risk. This narrative review consolidates current evidence on the epidemiology and neurobiological foundations of sleep disturbances in the psychosis spectrum and critically evaluates available non-pharmacological and digital interventions aimed at targeting sleep as a modifiable clinical outcome. We posit that sleep represents a critical, potentially modifiable intervention target within psychosis and that integrating sleep-focused care into standard clinical pathways may substantially enhance clinical, functional, and safety outcomes throughout the illness spectrum, pending replication in adequately powered randomized controlled trials.

## 1. Introduction

Sleep disturbances constitute one of the most consistent and clinically significant findings within the psychosis spectrum. From the earliest prodromal stages, including the clinical high-risk (CHR) mental states, to first-episode psychosis (FEP) and established schizophrenia, disrupted sleep remains a nearly universal characteristic that has historically been dismissed as a secondary consequence of psychiatric illness or antipsychotic medication [1,2]. Increasing evidence now challenges this perspective, e.g., sleep problems in psychosis are prevalent, diverse, and bidirectionally associated with the core features of the disorder, such as positive symptoms, negative symptoms, cognitive impairment, depression, and suicidal behavior [3,4].

Epidemiological studies consistently report that the prevalence of clinically significant insomnia exceeds 50–80% among individuals with psychosis spectrum disorders, with multiple comorbid sleep disorders, including nightmare disorder, sleep apnea, hypersomnia, and circadian rhythm sleep–wake disorders, frequently co-occurring within the same patient [5]. A systematic review and meta-analysis conducted by Bagautdinova and colleagues [6], which includes data from CHR, early psychosis, and chronic psychosis populations, demonstrated that sleep disturbances are widespread throughout the course of the illness, exhibiting common abnormalities in subjective sleep quality alongside distinct, stage-specific profiles of objective sleep architecture dysfunction. Importantly, alterations in sleep spindle activity, slow-wave sleep, and circadian entrainment are observable even in individuals who are drug-naïve at the onset of psychosis, suggesting that these disruptions are indicative of underlying neurobiological vulnerabilities rather than solely attributable to pharmacological effects [6,7].

The clinical significance of sleep disturbances in the psychosis spectrum extends far beyond subjective discomfort. Disturbed sleep is associated with greater severity of positive symptoms, particularly delusions and hallucinations, as well as with depression, anxiety, cognitive impairment, and reduced quality of life [8,9]. The relationship is bidirectional, i.e., not only do psychotic symptoms disrupt sleep, but experimental sleep deprivation reliably induces psychotic-like experiences in healthy individuals, lending causal plausibility to the hypothesis that sleep disruption actively contributes to the expression of psychosis [10]. Insomnia has been identified as a significant risk factor for suicidal ideation and behavior in FEP and CHR populations, an association that warrants consideration alongside comorbid depression, anxiety, and psychotic symptom severity as potential contributing factors [11,12].

Despite the substantial prevalence of sleep disturbances within the psychosis population, formal sleep assessments remain infrequent in routine psychiatric practice. Moreover, sleep interventions are generally regarded as ancillary to the management and limited to the use of hypnotic medications, while evidence-based interventions are seldom provided [13]. Cognitive Behavioral Therapy for Insomnia (CBT-I), the globally endorsed first-line treatment for insomnia, has begun to be tailored for individuals experiencing psychotic symptoms; however, its adoption has been sluggish [14,15]. Innovative digital health technologies present a potentially transformative avenue to broadly deliver evidence-based sleep interventions to this clinical population [16,17]. Digital interventions may be particularly suited to patients with psychosis for several reasons, including the barriers that often limit access to traditional face-to-face care, the practical advantages of asynchronous tools for individuals with irregular sleep–wake patterns, and the relatively high digital literacy observed among young people at early stages of psychosis. Furthermore, smartphone-based platforms offer the potential for continuous, ecologically valid monitoring of sleep, complementing or extending what single-point clinical assessments can capture.

This narrative review aims to synthesize evidence on sleep disturbances across the psychosis spectrum, elucidate neurobiological mechanisms linking disrupted sleep to psychotic symptoms, critically appraise evidence for non-pharmacological and digital sleep interventions, address barriers to implementation, and offer evidence-informed clinical recommendations. We draw on recent systematic reviews, randomized controlled trials, and original data from our research group to provide an integrative and clinically actionable perspective.

## 2. Materials and Methods

### 2.1. Search Strategy

A systematic literature search was conducted in February 2026 across four main electronic databases: PubMed/MEDLINE, Web of Science, EMBASE, and PsycINFO. The search combined terms for sleep (“sleep”, “insomnia”, “sleep disturbances”, “sleep quality”, “circadian rhythm”, “sleep architecture”, “nightmare”, “actigraphy”, “polysomnography”) with terms for psychosis (“psychosis”, “schizophrenia”, “first-episode psychosis”, “clinical high-risk”, “ultra-high risk”, “early psychosis”, “schizoaffective”) and terms for intervention (“cognitive behavioral therapy”, “CBT-I”, “digital”, “app”, “smartphone”, “wearable”, “light therapy”, “chronotherapy”, “mindfulness”, “image rehearsal therapy”, “non-pharmacological”). Reference lists of included studies and relevant review articles were manually searched. No date restriction was applied to the mechanistic and epidemiological literature. For intervention studies, the search was limited to publications from January 2010 onward. The search was updated in March 2026.

### 2.2. Inclusion and Exclusion Criteria

For inclusion, studies were required (1) to include adult participants (aged ≥ 18 years) with a diagnosis of schizophrenia spectrum disorder, FEP, bipolar disorder with psychotic features, or a CHR/ultra-high-risk (UHR) state; (2) to address sleep disturbances as a primary or secondary outcome, an exposure variable, or a mechanistic mediator; and (3) to be published in peer-reviewed English-language journals. For interventional sections, only studies using quantitative outcome measures for sleep and/or psychopathology were included. Eligible designs included RCTs, non-randomized controlled trials, feasibility and pilot studies, cohort studies, cross-sectional studies, and systematic reviews with or without meta-analyses. Case reports, conference abstracts, and studies focusing exclusively on pharmacological sleep interventions were excluded.

### 2.3. Study Selection and Data Synthesis

Two authors (V.B. and M.G.) independently screened titles and abstracts and conducted full-text reviews of potentially eligible records. Disagreements were resolved by consensus with a third senior author (G.P.). Given the narrative format and substantial heterogeneity in study designs, populations, interventions, and outcome measures, a formal meta-analytic synthesis was not performed. Findings are synthesized thematically, with attention to evidence quality, effect sizes, and clinical applicability. Key intervention studies are summarized in Table 1. Although the search process incorporated systematic elements, including predefined search terms, multiple databases, explicit inclusion and exclusion criteria, and dual independent screening, this review does not constitute a systematic review. No PRISMA flow diagram, formal risk-of-bias assessment, or quality grading of individual studies was conducted. The structured search was adopted to minimize unsystematic source selection and to enhance the reproducibility of the literature base, consistent with SANRA recommendations for rigorous narrative reviews. The synthesis remains thematic and narrative in nature, and findings should be interpreted accordingly.

This review was conducted in accordance with the Scale for the Assessment of Narrative Review Articles (SANRA) guidelines [18].

## 3. Results

### 3.1. Prevalence and Phenomenology of Sleep Disturbances Across the Psychosis Spectrum

The prevalence and phenomenological profile of sleep disturbances in the psychosis spectrum vary considerably across different diagnostic categories and stages of illness. However, clinically significant sleep disruption is nearly ubiquitous throughout the illness course. Insomnia disorder, characterized by difficulty initiating or maintaining sleep, early morning awakening, or non-restorative sleep with substantial daytime repercussions, is the most frequently documented condition, with prevalence estimates ranging from 50% to 80% in both early and chronic psychosis populations [5,6]. Nightmare disorder, defined by recurrent distressing dreams that cause clinically significant distress or impairment, affects an estimated 30–60% of patients with schizophrenia spectrum disorders, a markedly higher rate compared to the general population (2–6%), and is often associated with a history of trauma, comorbid post-traumatic stress disorder (PTSD), and increased suicidal risk [5]. Hypersomnia, characterized by excessive daytime sleepiness despite adequate or extended nocturnal sleep, is disproportionately prevalent in individuals with schizophrenia relative to other psychiatric diagnoses, partly attributable to sedation induced by antipsychotic medication but also reflecting intrinsic illness-related alterations in arousal regulation [19].

Obstructive sleep apnea (OSA) is frequently overlooked in clinical settings for the treatment of psychosis. However, it is markedly more prevalent within this population compared to the general population [20]. This increased prevalence is driven by the combined effects of antipsychotic-related weight gain, metabolic syndrome, sedation, and potentially illness-related abnormalities in upper airway muscle tone [13]. Estimates of OSA prevalence in individuals with schizophrenia range from 15% to 48%, depending on the assessment methodology employed [20]. The underdiagnosis of OSA in individuals with psychosis reflects a systemic failure in recognition that is not sufficiently mitigated by standard screening instruments. Spontaneous reporting of OSA symptoms is frequently suppressed by antipsychotic sedation, negative symptoms, social isolation, and limited insight.

Commonly employed OSA screening tools, such as the STOP-BANG questionnaire and the Epworth Sleepiness Scale, have not undergone validation within psychosis populations and are therefore likely to underperform in this context. This underscores the need to maintain a lower threshold for objective investigative procedures, particularly polysomnography or home sleep apnea testing, in patients presenting with unexplained residual cognitive deficits, treatment-refractory positive symptoms, or excessive daytime sleepiness that are not fully explained by medication. The pathophysiological consequences of untreated OSA in psychosis are particularly concerning because they directly compound the neurobiological vulnerabilities already characteristic of the disorder. Continuous positive airway pressure (CPAP) therapy remains the primary treatment for moderate-to-severe OSA. However, adherence within psychosis populations presents specific clinical challenges that are not sufficiently addressed by conventional CPAP initiation protocols. Factors such as claustrophobia, nocturnal hypervigilance, paranoid ideation exacerbated by mask application, and cognitive difficulties in learning device management can collectively diminish the efficacy of standard procedures. Undiagnosed and untreated OSA significantly contributes to daytime cognitive impairments, treatment-refractory psychotic symptoms, and cardiometabolic morbidity.

Finally, circadian rhythm sleep–wake disorders (CRSWDs), which include delayed sleep–wake phase disorder, irregular sleep–wake rhythm disorder, and non-24 h sleep–wake rhythm disorder, represent a distinct and often underrecognized sleep phenotype in psychosis, necessitating specific assessment and treatment strategies that differ substantially from those used for insomnia disorder [20,21].

The distribution of these sleep disorders across the psychosis spectrum is summarized in Table 1.

### 3.2. Clinical High-Risk Mental States

Individuals at CHR for psychosis demonstrate rates of sleep disturbance that are comparable to, and in some instances surpass, those observed in established psychotic disorders [6,22]. A systematic review and meta-analysis conducted by Clarke and colleagues [22] identified significantly increased rates of insomnia, poor sleep quality, and circadian irregularities among CHR individuals compared to healthy controls, with prevalence estimates for clinically significant insomnia ranging from 50% to 75%.

The bidirectional and temporally dynamic nature of the relationship between sleep and psychosis is particularly well-characterized within CHR populations. A study by Formica and coworkers [23] analyzed daily associations between sleep parameters and attenuated psychotic symptoms in 76 CHR young individuals over six days of intensive Experience Sampling Methodology (ESM), demonstrating that nightly sleep fragmentation, operationalized as the number of nocturnal awakenings, unidirectionally predicted increased suspiciousness and feelings of unreality the following day, even after controlling for negative affect and substance use. Longitudinal actigraphic data from Lunsford-Avery further indicated that baseline sleep disruption in adolescents at clinical high risk forecasted increased severity of positive symptoms at a 12-month follow-up, thereby raising the possibility that sleep disruption serves as a temporally antecedent, potentially modifiable risk marker for the progression of psychosis [24].

### 3.3. First-Episode Psychosis

In individuals experiencing FEP, sleep disturbances are widespread, complex, and often remain untreated. A systematic review comprising 1255 patients with FEP and 342 healthy controls identified the most consistently reported objective abnormalities as increased sleep latency and reductions in slow-wave sleep (SWS), along with heightened frequencies of sleep spindle deficits observed in polysomnography studies [3]. Evidence of bidirectional relationships between sleep disturbances and psychotic symptoms was documented across various study designs, with insomnia severity positively correlating with the severity of positive symptoms, depression, and functional impairment.

The scope of sleep pathology in FEP extends beyond general poor sleep quality to encompass a wide range of diagnosable sleep disorders. A study assessing 60 outpatients aged 18 to 30 with non-affective psychosis found that 8% met criteria for at least one sleep disorder, with insomnia and nightmare disorder being the most prevalent. Comorbidity was notably high, averaging 3.3 sleep disorders per patient. Despite their clinical significance, over half of the identified disorders had been discussed with a clinician, yet nearly 75% had received no formal treatment [5], underscoring a critical gap between recognition and therapeutic intervention. These disturbances are not confined solely to subjective experience, but they are also reflected in objective neurophysiological measures. High-density EEG recordings in FEP patients have demonstrated significant reductions in sleep spindle duration and density compared to healthy controls, predominantly in frontal regions. These deficits specifically correlate with the severity of negative symptoms [25]. Complementing this, polysomnographic studies in drug-naive FEP patients revealed a broad profile of cognitive impairment, including verbal memory, processing speed, and working memory, associated with disrupted sleep architecture and increased negative symptom burden [26]. This evidence suggests that thalamocortical dysfunction underlying spindle generation may serve as a neurobiological interface between sleep pathology and cognitive decline in early psychosis.

Furthermore, a cross-sectional study involving 11 FEP patients within 12 months from the onset of psychosis and matched healthy controls demonstrated that a majority of patients experienced poor sleep quality (70%), depressive symptoms (60%), and suicidal ideation (65%). Subjective sleep quality, measured by the Pittsburgh Quality Index (PSQI), exhibited a strong correlation with depressive and prodromal psychotic symptoms. Actigraphic assessments also revealed that patients had significantly reduced total sleep time compared to controls [4].

### 3.4. Chronic Psychosis

In established schizophrenia, sleep disturbances endure despite pharmacological stabilization and frequently constitute one of the most disabling residual symptoms of the disorder. Wulff and colleagues [19] conducted a seminal six-week study using actigraphy and melatonin profiling in 12 stable outpatients with schizophrenia and 31 unemployed healthy controls [19]. Severe disruptions in sleep and circadian rhythms were observed in all participants with schizophrenia. Specifically, roughly half of them demonstrated pronounced circadian misalignment, including phase-delayed, phase-advanced, and non-24 h sleep–wake cycles accompanied by abnormal melatonin profiles, while the remaining exhibited markedly irregular and fragmented sleep patterns despite relatively preserved melatonin phase timing. The average sleep duration in the schizophrenia cohort exceeded that of controls by more than 2 h, with significantly delayed sleep offset, substantially reduced daily bright-light exposure—a critical factor in circadian regulation—and increased variability in sleep timing from day to day. These findings highlight the considerable heterogeneity of sleep phenotypes observed in chronic schizophrenia and emphasize the inadequacy of generalized intervention strategies, thereby advocating for individualized, phenotype-driven sleep treatment approaches.

Beyond gross architectural disturbances, chronic schizophrenia is characterized by a highly specific, neurobiologically informative deficit in sleep spindles—brief 12–16 Hz thalamocortical oscillations during NREM stage 2 sleep that mediate memory consolidation and reflect the integrity of underlying thalamo-reticular circuits. High-density EEG studies have consistently shown marked reductions in spindle density, amplitude, and duration in patients with schizophrenia compared to healthy controls and other psychiatric comparison groups, such as individuals with a history of depression [27]. Critically, the spindle deficit in schizophrenia correlates with impaired sleep-dependent memory consolidation, the severity of positive symptoms, and abnormal thalamocortical connectivity, pointing to dysfunction of the thalamic reticular nucleus (TRN), which generates spindles, gates the relay of sensory information to the cortex, and modulates thalamocortical communication. These spindle abnormalities are not attributable to antipsychotic medication alone, as they have been documented in antipsychotic-naive early-course patients and in non-psychotic first-degree relatives, suggesting that reduced spindle activity may represent a heritable endophenotype of the schizophrenia spectrum [28]. The functional implications of this spindle deficit extend to sleep-dependent memory consolidation, an offline cognitive process notably impaired in individuals with schizophrenia. A systematic review and meta-analysis in this domain indicated that, while sleep significantly enhances procedural memory consolidation in healthy adults, the effect observed in individuals with schizophrenia is modest, with a moderate between-group difference [29]. Additionally, a qualitative review similarly identified deficits in sleep-dependent declarative memory consolidation among patients compared to healthy controls. These impairments are directly pertinent to functional outcomes, as cognitive deficits, including memory, constitute the most robust predictors of disability in schizophrenia.

These neurophysiological findings are further contextualized by large-scale epidemiological data. Poor sleep quality in schizophrenia is associated with relapse risk, accelerated cognitive decline, and worsened social functioning, thereby increasing barriers to effective treatment and rehabilitation [30]. Rates of poor sleep quality, as measured by the PSQI, vary substantially across studies (30–80%), with discrepancies likely attributable to differences in symptom severity, cognitive functioning, medication regimens, and threshold criteria [30]. Taken together, these findings reinforce a model in which sleep disturbances in chronic schizophrenia are not epiphenomenal but constitute an active neurobiological mechanism sustaining cognitive impairment and symptom burden, and a potentially modifiable treatment target if interventions can be designed to restore not merely the quantity but the oscillatory architecture of sleep.

## 4. Neurobiological Mechanisms Linking Sleep and Psychosis

**Figure 1:**
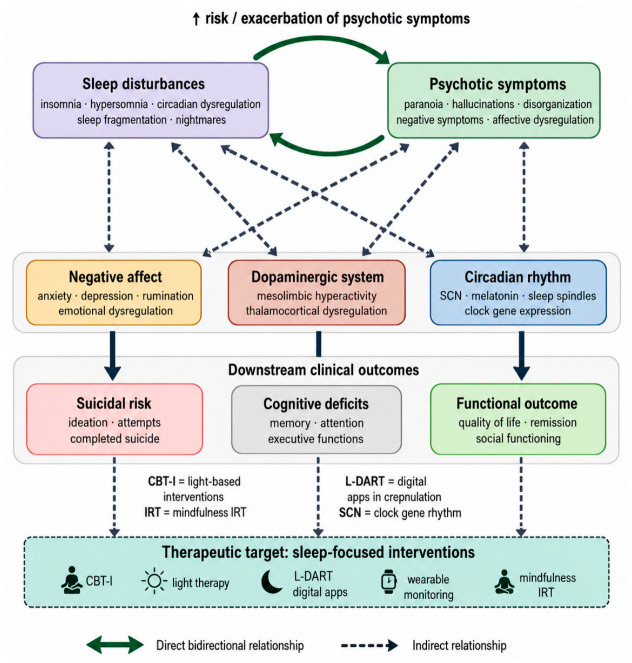
Schematic representation of the bidirectional mechanisms linking sleep disturbances and psychotic symptoms across the psychosis spectrum. Direct bidirectional relationships (solid arrows) and mediating pathways (dashed arrows) through negative affect, dopaminergic dysregulation, and circadian rhythm disruption are shown. Downstream clinical outcomes—suicidal risk, cognitive deficits, and functional impairment—are modulated by the sleep–psychosis interaction. Sleep-focused interventions (bottom panel) represent a multi-entry-point therapeutic target addressing multiple nodes in this network. CBT-I = Cognitive Behavioral Therapy for Insomnia; IRT = Image Rehearsal Therapy; L-DART = Light-Dark and Activity Rhythm Therapy; SCN = suprachiasmatic nucleus.

A stepped-care framework for sleep intervention in psychosis, wherein treatment intensity is calibrated according to clinical complexity and prior treatment response, embodies a rational and resource-efficient implementation strategy. In this model, the initial stage involves concise universal sleep psychoeducation and fundamental stimulus control guidance, which can be provided by any trained clinician during routine consultations. The subsequent stage incorporates a digital or group-based CBT-I program targeting patients experiencing persistent, clinically significant insomnia following the initial intervention. The third stage entails individually tailored, face-to-face CBT-I administered by a specialist clinician, with the capacity to incorporate nightmare-specific treatments, circadian rhythm interventions, or assessment for OSA as clinically warranted. This framework optimizes population coverage while reserving intensive specialist resources for patients with the highest clinical complexity, aligning with the recommendations of the National Institute for Health and Care Excellence (NICE) and the American College of Physicians regarding insomnia management broadly. Figure 1 depicts this stepped-care model as applied to the psychosis spectrum.

The mechanisms by which sleep disturbances contribute to and are perpetuated by psychotic symptoms encompass molecular, circuit-level, and systems neuroscience perspectives. Figure 1 offers a schematic representation of the principal bidirectional pathways identified in the literature, including direct relationships between sleep and psychotic symptoms, as well as mediating pathways through negative affect, dopaminergic dysregulation, and circadian rhythm disruption. Three primary mechanistic domains merit detailed examination.

### 4.1. Thalamocortical Dysregulation and Sleep Spindle Deficits

Sleep spindles—bursts of rhythmic oscillatory activity at 12–15 Hz generated by thalamocortical circuits during NREM sleep—have emerged as a compelling neurophysiological marker shared by sleep and psychosis pathology. Manoach and Stickgold reviewed converging evidence demonstrating that individuals with schizophrenia exhibit significant deficits in sleep spindle density, amplitude, and duration relative to healthy controls, detectable in drug-naive patients and therefore not attributable to antipsychotic treatment alone [7]. Sleep spindle generation depends on the integrity of the thalamic TRN and its GABAergic projections to thalamocortical neurons, precisely the circuitry implicated in sensory gating, attentional filtering, and the thalamocortical dysconnectivity thought to underlie positive symptoms and cognitive impairment in schizophrenia. In the healthy brain, the TRN acts as a gating structure that regulates the relay of sensory information to the cortex, suppressing irrelevant inputs during sleep and waking alike. Dysfunction within this circuit not only impairs spindle generation but is hypothesized to result in a failure of sensory filtering that may directly contribute to the emergence of hallucinations and other positive symptoms, establishing a mechanistic continuum between sleep neurophysiology and waking psychopathology [31].

During NREM sleep, spindles do not act in isolation but coordinate with cortical slow oscillations and hippocampal sharp-wave ripples to mediate the offline transfer of newly encoded memories from hippocampal to neocortical storage, a process known as systems consolidation. Critically, it is not spindle density per se, but the precise temporal coupling of spindles with slow oscillations that predicts overnight memory improvement. This distinction carries direct therapeutic implications: a double-blind, crossover randomized controlled trial by Mylonas et al. demonstrated that while eszopiclone significantly increased spindle density in medicated schizophrenia patients, it failed to improve procedural memory consolidation, because the drug simultaneously disrupted slow oscillation morphology and reduced the phase-locking consistency between spindles and slow oscillations [32]. These findings suggest that effective interventions targeting sleep-dependent cognition in schizophrenia must restore the coordinated oscillatory dialogue between thalamic and cortical structures, rather than merely augmenting spindle quantity in isolation.

Spindle deficits in schizophrenia have been associated with impaired overnight memory consolidation, contributing to the well-documented episodic and procedural memory deficits characteristic of the disorder [7]. Reduced spindle activity has additionally been correlated with greater severity of positive symptoms, implying that TRN dysfunction may concurrently diminish memory consolidation during sleep and heighten psychotic symptomatology during wakefulness [28]. Supporting this mechanistic hypothesis, spindle activity demonstrates high heritability, exceeding 80% in twin studies, and has been linked to schizophrenia risk genes, most notably *CACNA1I*, which encodes a T-type calcium channel subunit essential for TRN burst firing and spindle initiation. The knockout of this gene in animal models results in spindle deficits that phenotypically resemble those observed in schizophrenia, positioning it as a potential molecular bridge between genetic risk factors and neurophysiological abnormalities [33]. Collectively, these findings endorse reduced sleep spindle activity as a heritable endophenotype within the schizophrenia spectrum that reflects TRN-mediated thalamocortical circuit dysfunction, thereby contributing to both cognitive impairments and the severity of positive symptoms. Furthermore, they identify GABAergic and calcium channel signaling within the TRN as promising targets for the development of novel therapeutic interventions.

This mechanistic account predicts that interventions capable of genuinely enhancing the coordinated oscillatory architecture of NREM sleep, whether through pharmacological modulation of TRN circuitry, closed-loop auditory stimulation timed to slow oscillation up-states, or behavioral approaches such as sleep restriction within CBT-I that increase homeostatic sleep pressure and may augment NREM depth, could simultaneously improve sleep quality, memory consolidation, and attenuate psychotic symptoms.

### 4.2. Dopaminergic Dysregulation and Sleep Wake Control

Mesolimbic dopamine hyperactivity, the most robust neurochemical finding in schizophrenia, drives aberrant salience attribution and underlies positive symptoms. Additionally, dopaminergic projections modulate key sleep-regulating structures, including the ventral tegmental area, locus coeruleus, and basal forebrain [25]. Excess dopaminergic tone promotes wakefulness and suppresses REM sleep, aligning with frequent observations of prolonged sleep latency, decreased sleep efficiency, and disturbed REM sleep during acute psychosis. Conversely, antipsychotic medications that antagonize D2 receptors consistently improve sleep continuity and diminish sleep latency, thereby providing pharmacological evidence for the causal role of dopaminergic hyperactivity in sustaining sleep disturbances associated with the disorder. Nevertheless, their effects on sleep architecture, particularly on SWS and spindle activity, are variable and sometimes paradoxical [34].

The relationship between sleep deprivation and dopaminergic signaling has been directly characterized using PET neuroimaging. Volkow et al. demonstrated that a single night of total sleep deprivation in healthy volunteers significantly reduced striatal binding of [^11^C]raclopride, a radiotracer that competes with endogenous dopamine for D2/D3 receptor binding, interpreted as evidence of increased dopamine release in the striatum [35]. A subsequent study by the same group further showed that these changes in striatal dopamine were associated with impaired visual attention performance and aberrant activation patterns in dopaminergically modulated cortical regions, including the anterior cingulate and insula [36], providing a mechanistic link between sleep loss, striatal hyperdopaminergia, and the attentional and perceptual dysregulation characteristic of psychosis.

These neuroimaging findings align with experimental evidence derived from translational paradigms. Sleep deprivation in healthy volunteers consistently causes deficits in prepulse inhibition (PPI) of the acoustic startle response, a well-validated cross-species biomarker of sensorimotor gating that is frequently impaired in individuals with schizophrenia, in addition to self-reported perceptual distortions, cognitive disorganization, and anhedonia [37]. Importantly, PPI deficits caused by sleep deprivation in rodents are selectively mitigated by antipsychotic medications but not by anxiolytics or antidepressants, thereby providing pharmacological specificity to the model and affirming its validity as a translational surrogate for psychotic states [37]. Furthermore, a review of converging evidence indicates that experimentally controlled sleep deprivation results in a dose-dependent spectrum of abnormalities, encompassing positive, negative, and cognitive symptom domains, along with impairments in various translational biomarkers, including smooth pursuit eye movements and antisaccades [38]. This supports the use of sleep deprivation as a reproducible experimental medicine model of psychosis.

### 4.3. Circadian Rhythm Disruption and the Sleep-Circadian Interface

The suprachiasmatic nucleus (SCN) of the hypothalamus coordinates 24 h rhythms in sleep–wake patterns, melatonin secretion, cortisol release, and core body temperature through a network of molecular clock genes [20,25]. In cases of schizophrenia, convergent evidence from post-mortem studies, cellular models, actigraphic analyses, and melatonin profiling suggests extensive disruption of circadian organization at both molecular and behavioral levels [18]. At the cellular level, Johansson et al. demonstrated that fibroblasts cultured from individuals with chronic schizophrenia exhibited a loss of rhythmic expression of *CRY1* and *PER2* in comparison to cells from healthy controls, while mononuclear blood cells from first-episode patients displayed decreased expression of several core clock genes [39], providing direct evidence that molecular clock dysfunction is innate to the disorder and manifests from its earliest stages. This molecular disruption is further exacerbated by dopaminergic dysregulation: the *CLOCK* gene T3111C polymorphism has been associated with schizophrenia and linked to abnormal dopaminergic transmission to the SCN, while D2 receptor signaling appears to influence CLOCK:BMAL1 transcriptional activity [40], establishing a bidirectional interface between the circadian and dopaminergic systems that may intensify dysregulation in both.

A critical downstream consequence of circadian disruption in schizophrenia is the attenuation of nocturnal melatonin secretion. Studies involving drug-free patients with chronic schizophrenia have consistently demonstrated a significant reduction in nocturnal plasma melatonin levels compared to healthy controls, a deficit that persists despite antipsychotic treatment and is partially attributable to decreased pineal gland volume and increased pineal calcification [41].

Meyer et al. further articulated the sleep-circadian interface as a transdiagnostic window into mental disorders, noting that disruption to either the circadian pacemaker or homeostatic sleep pressure can precipitate or exacerbate psychiatric symptoms across diagnostic boundaries. Taken together, the evidence reviewed in this section positions circadian disruption in schizophrenia not as an epiphenomenon of irregular lifestyle but as a neurobiologically grounded feature of the illness, rooted in molecular clock dysfunction, pineal hypoactivity, reduced photic entrainment, and dopaminergic dysregulation, that warrants targeted chronotherapeutic intervention alongside standard pharmacological treatment.

## 5. Non-Pharmacological Interventions for Sleep in Psychosis

### 5.1. Cognitive Behavioral Therapy for Insomnia: Individual and Group Formats

**Figure 2:**
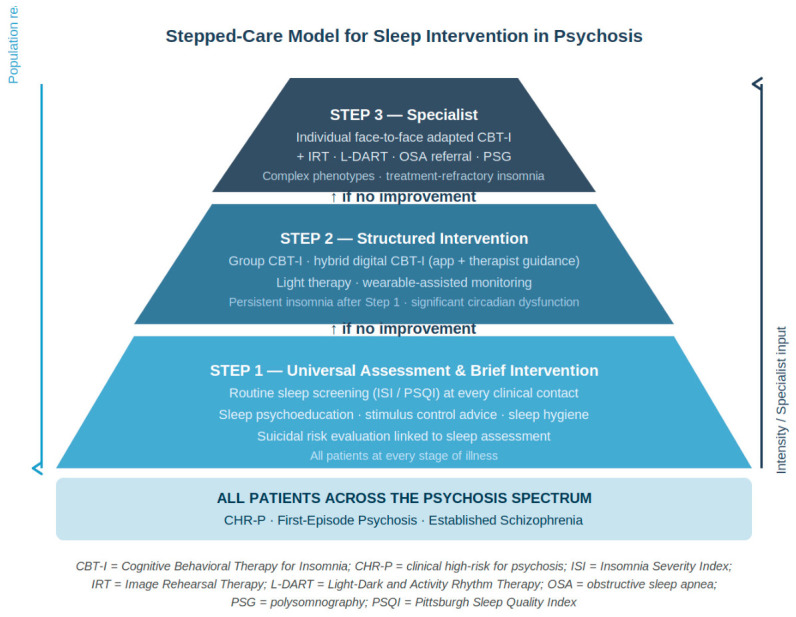
Stepped-care model for the assessment and management of sleep disturbances in psychosis. Treatment intensity increases across steps while population reach decreases. Step 1 is universal and applicable to all patients at every stage of illness. Step-up decisions are guided by clinical response to prior intervention. CBT-I = Cognitive Behavioral Therapy for Insomnia; CHR-P = clinical high-risk for psychosis; ISI = Insomnia Severity Index; IRT = Image Rehearsal Therapy; L-DART = Light-Dark and Activity Rhythm Therapy; OSA = obstructive sleep apnea; PSG = polysomnography; PSQI = Pittsburgh Sleep Quality Index.

Figure 2 reports the stepped-care model for the assessment and management of sleep disturbances in psychosis. CBT-I is internationally recommended as the first-line treatment for chronic insomnia disorder, with demonstrated superiority over pharmacotherapy in long-term efficacy and durability [42,43]. Meta-analytic evidence from 87 RCTs in general population samples demonstrates large effect sizes on self-reported insomnia severity (Cohen’s d ≈ 1.0) and moderate effects on objective actigraphic parameters [14]. Core CBT-I components comprise sleep restriction therapy, stimulus control, sleep hygiene education, cognitive restructuring of dysfunctional beliefs and attitudes regarding sleep, and relaxation techniques. Psychosis-specific adaptations include the implementation of modified sleep restriction protocols applied more gradually with enhanced symptom monitoring, cognitive restructuring techniques tailored for delusional thinking or disorganized cognition, simplified psychoeducational content with scaffolding to accommodate cognitive impairments, and integration within an established therapeutic relationship with a familiar clinician to enhance engagement.

Group-format CBT-I, wherein the intervention is administered to small cohorts of four to eight participants concurrently, offers a pragmatic compromise between the accessibility of digital modalities and the guided quality characteristic of individual therapy. Empirical evidence indicates that group CBT-I exhibits efficacy comparable to that of individual treatment within general population samples and provides additional therapeutic benefits, including the normalization of insomnia experiences, peer support, and social accountability for behavioral modifications. Within the framework of psychosis services, CBT-I has been piloted in early intervention initiatives and community mental health teams. Preliminary results indicate its feasibility and acceptability even among patients presenting with substantial residual positive and negative symptoms [44]. The Finnish randomized controlled trial currently underway, conducted by Tanskanen, directly compares internet-delivered and group-format CBT-I with treatment as usual in outpatients diagnosed with schizophrenia or schizoaffective disorder, with recruitment targeting 84–120 participants from the Psychosis Clinics of Helsinki University Hospital [45]. The published protocol represents the first rigorous, pre-registered comparative design of these delivery modalities in a clinical psychosis population; however, results are not yet available, with full interpretation of the findings expected in approximately 2028.

A systematic review and meta-analysis examining CBT-I in patients with psychotic disorders, encompassing eight eligible studies, found significant improvements in insomnia and sleep quality in both the short and long term, alongside significant short-term improvements in psychotic symptoms and mental well-being [46].

### 5.2. Sleep Interventions in Clinical High-Risk and Early Psychosis Populations

The SleepWell program represents one of the most clinically significant recent advances in sleep intervention research at the early stages of the psychosis continuum. Developed and tested by Waite, Freeman, and colleagues at the University of Oxford [47], SleepWell is a brief psychological intervention delivered in approximately eight individual one-hour sessions over 12 weeks, targeting young people aged 14–25 who are at ultra-high risk of psychosis and present with clinically significant insomnia. The intervention is based on standard CBT-I protocols and established sleep treatments tailored for individuals with psychosis, featuring specific adaptations for the CHR population. Core therapeutic components comprise stimulus control, circadian realignment, sleep pressure regulation through daytime activity, and strategies to reduce hyperarousal, including worry reduction techniques and cognitive restructuring. Furthermore, additional modules address the unique contextual factors characteristic of this age group, such as adolescent-specific biological changes in sleep architecture, delayed circadian phase, environmental constraints, and the relationship between psychotic-like experiences and sleep disruption [48].

In a randomized controlled feasibility trial involving 40 young individuals at ultra-high risk of psychosis, SleepWell exhibited exceptionally high treatment acceptability, with a median score of 48 out of a maximum possible 48 on the Abbreviated Acceptability Rating Profile, and yielded clinically significant, sustained reductions in insomnia severity at both three months and nine months post-randomization compared to usual care alone. Preliminary benefits were also observed regarding psychotic experiences and depressive symptoms [47]. Notably, no participant in the SleepWell group transitioned to psychosis during the trial period, whereas one participant in the usual care group did. This observation must be interpreted with considerable caution: the study was not powered to assess psychosis transition as an endpoint, the difference involves only a single event, and no causal inference regarding prevention of psychosis onset can be drawn from this finding [47].

These preliminary findings are consistent with the hypothesis that sleep disturbance may not be solely a prodromal symptom but also a potentially modifiable factor in the trajectory toward psychosis. Whether intervening on sleep prior to illness onset constitutes a genuinely preventive approach remains to be established in adequately powered prospective trials with psychosis transition as a primary endpoint. Complementing this research within the CHR population, the CRISP trial examined the feasibility and acceptability of digital CBT-I (Sleepio) in a service for FEP. This trial enrolled individuals experiencing a first episode of psychosis with clinically significant insomnia [44]. The study extends the evidence base for sleep interventions from the at-risk phase into early psychosis, supporting the development of a staged, clinically integrated sleep intervention model that spans the entire early psychosis continuum, ranging from UHR to FEP, and provides an empirical foundation for integrating sleep-focused psychological treatment within standard early intervention services for psychosis.

### 5.3. Circadian-Focused Interventions: Light Therapy and L-DART

Light therapy (2500–10,000 lux, 20–60 min daily, administered in the morning for phase-delay disorders) is well-established for circadian sleep–wake disorders and seasonal affective disorder. A prospective cohort study involving 20 inpatients with schizophrenia evaluated a dynamic circadian rhythm simulation lighting (CRSL) system over a period of 10 weeks. The study observed improvements in psychopathology, as measured by the BPRS total score, and cognitive function, as assessed by the MMSE, with no adverse events attributable to the light intervention [48].

Light-Dark and Activity Rhythm Therapy (L-DART) is a comprehensive behavioral intervention developed at the University of Oxford, designed to address the heterogeneous sleep phenotypes of schizophrenia spectrum disorders [49]. Delivered by an occupational therapist over 12 weeks, L-DART combines an individualized light-exposure prescription, physical activity scheduling, behavioral sleep strategies adapted from CBT-I, and environmental modifications to optimize the home light-dark cycle. A mixed-methods feasibility study demonstrated high acceptability, satisfactory retention, and preliminary improvements in subjective sleep and well-being across participants with insomnia, hypersomnia, irregular sleep–wake timing, and non-24 h cycles [21]. A key strength of L-DART is its accommodation of the full phenotypic spectrum of sleep disturbances in schizophrenia within a single, personalizable protocol, distinguishing it from CBT-I, which targets insomnia specifically and requires substantial adaptation for patients with hypersomnia or circadian timing disorders.

### 5.4. Mindfulness-Based Interventions and Image Rehearsal Therapy

Mindfulness-Based Stress Reduction (MBSR) and Mindfulness-Based Cognitive Therapy (MBCT) have demonstrated efficacy for insomnia and related sleep disturbances in general and clinical psychiatric populations, operating through reduced pre-sleep cognitive and somatic arousal, enhanced acceptance of wakefulness-related distress, and improved emotion regulation [50]. Adaptations of MBIs for psychosis (MBCTp) have demonstrated safety and feasibility, although their specific effects on sleep quality in psychosis have not been systematically evaluated. Their emphasis on non-judgmental acceptance may be particularly suited to patients with psychosis experiencing hypervigilance and anxiety-driven insomnia.

Image Rehearsal Therapy (IRT) specifically targets nightmare disorder through cognitive rehearsal of a rescripted, less threatening version of a recurrent nightmare during wakefulness, reducing nightmare frequency and distress through repetition-based reconsolidation mechanisms. Nightmare disorder affects 30–60% of patients with schizophrenia spectrum disorders and contributes independently to sleep fragmentation, distress, and suicidal risk [51]. In a cross-sectional study of 40 patients with psychosis, Sheaves et al. found that 55% reported weekly distressing nightmares, with nightmare distress significantly correlated with delusional severity, depression, anxiety, and working memory impairment [52]. Building on this, the NITES trial tested a four-week CBT for nightmares intervention incorporating IRT as the primary technique in 24 patients with persecutory delusions. The trial demonstrated large effect sizes for improvements in nightmare severity and insomnia at post-treatment and follow-up, with a medium effect size benefit for paranoia, though the confidence intervals were wide, and a larger trial was deemed necessary [52]. An earlier case series by the same group had already established the acceptability and feasibility of adapting IRT for individuals with co-occurring psychotic symptoms, reporting descriptive improvements in nightmare-related distress, vividness, and intensity across five participants [53].

The clinical relevance of IRT in psychosis is further underscored by the high prevalence of trauma and comorbid PTSD in this population. A systematic review and meta-analysis found a current prevalence of comorbid PTSD in schizophrenia spectrum disorders of 10.6%, rising to 30% in FEP [54,55], while rates of clinically significant trauma exposure exceed 80% in some clinical cohorts. Nightmare disorder in psychosis is not simply a proxy for PTSD: a large general population study confirmed a direct association between nightmares and both paranoia and hallucinatory experiences even after controlling for PTSD symptoms, suggesting that nightmares constitute an independent causal pathway to psychotic experiences rather than merely a downstream consequence of trauma [56]. Integration of IRT within broader sleep intervention protocols for psychosis, particularly in patients with co-occurring PTSD or significant trauma histories, therefore warrants systematic evaluation in adequately powered trials.

Table 2 reports the key of non-pharmacological interventions.

## 6. Digital Interventions for Sleep in Psychosis

### 6.1. Digital Delivery of CBT-I

The digital delivery of CBT-I has transformed access to evidence-based sleep treatment for the general population. Systematic reviews of fully automated digital CBT-I (dCBT-I) consistently show moderate-to-large effects on insomnia severity compared with control conditions, with treatment completion rates of 60–80% and effect sizes somewhat lower than those achieved by face-to-face therapist-delivered CBT-I [17]. Programs such as SleepioRx, cleared by the U.S. FDA as a prescription digital therapeutic for insomnia, deliver the full CBT-I protocol through interactive digital sessions personalized using daily sleep diary entries, without a human coaching component. Virtually all large-scale dCBT-I trials have explicitly excluded participants with psychotic disorders as standard exclusion criteria, limiting generalizability to these populations [59].

Illness-specific barriers, including cognitive impairments, negative symptoms, and potential difficulties with psychoeducational content, suggest that a degree of human guidance may be essential for this population. A feasibility study evaluated a seven-module smartphone-based CBT for sleep, delivered with weekly therapist contact, in 28 individuals with psychosis spectrum diagnoses [57]. Recruitment and retention were acceptable, satisfaction was high, and the intervention was associated with significant ISI reductions and improvements in depression and quality of life. Qualitative analyses highlighted the value of smartphone accessibility and the importance of human guidance for maintaining motivation, supporting a hybrid digital–human delivery model as the most promising format for this population.

The deployment of digital tools among people with psychosis raises specific clinical and ethical considerations that must be explicitly addressed. Privacy and data security are particularly concerning, given the sensitivity of continuously collected behavioral and physiological data. Paranoid ideation may lead some patients to interpret monitoring devices or smartphone prompts as intrusive or threatening, potentially reducing engagement or exacerbating symptoms. Cognitive impairment and negative symptoms may limit the usability of interfaces designed for general populations. Digital exclusion—arising from limited access to smartphones, unreliable internet connectivity, or low digital literacy—risks compounding existing health inequalities. These factors collectively underscore that digital sleep interventions cannot be recommended for universal implementation in their current form. Hybrid models that combine digital platforms with clinician oversight represent the most clinically plausible and ethically responsible approach at the present stage of evidence.

Digital mental health tools more broadly have been proposed as scalable platforms for continuous monitoring, prevention, and early intervention across psychiatric populations [60].

It must be emphasized that virtually all evidence on digital CBT-I for psychosis comes from single-arm feasibility studies with small sample sizes, short follow-up periods, and no active control condition. The significant reductions in ISI and quality-of-life improvements reported should therefore be regarded as preliminary signals that warrant confirmation in adequately powered RCTs, rather than as evidence of established clinical efficacy.

### 6.2. Wearable Technology and Sensor-Augmented Monitoring

Wearable sensors offer uninterrupted and objective data concerning sleep–wake cycles, physical activity, and light exposure without necessitating active participation from the user. This approach effectively overcomes adherence challenges linked to sleep diary completion among individuals with cognitive impairments or negative symptoms. The ‘Sleep Catcher’ application integrates data from a wrist-worn fitness tracker and a bed-mounted motion sensor to autonomously generate nightly sleep diaries for individuals with schizophrenia enrolled in CBT-I, demonstrating precise sleep diary generation and high acceptability during beta testing [58].

Beyond facilitating treatment adherence, wearable actigraphy offers a continuous, passive window into the behavioral correlates of clinical status in schizophrenia. The Sleepsight platform—developed through iterative co-design with patients, clinicians, and bioinformaticians—demonstrated that extended use of wearable and mobile technologies is acceptable to community-dwelling individuals with schizophrenia, and proposed that continuous rest-activity profiling and heart rate variability monitoring may yield predictive, objective markers of clinical deterioration detectable in the early stages of impending relapse [61]. An observational study enrolling 40 outpatients with schizophrenia, in which participants were continuously monitored using commercial actigraphy devices and smartphones over four months, demonstrated high compliance with wearable use (>80% data coverage) and found that sleep and activity features derived from actigraphy predicted clinically rated symptom severity in elastic net regression models [62]. A recent narrative review of wearables in schizophrenia further confirmed that sleep disturbances captured by actigraphy are predictive of symptom exacerbation and acute worsening of psychotic symptoms, and that machine learning applied to wearable-derived heart rate and motor activity can discriminate patients from healthy controls, with wearables additionally capable of capturing autonomic dysregulation during active paranoia, hallucinations, and delusions [63].

These findings, while technologically promising, are derived predominantly from observational studies and small feasibility cohorts. Whether wearable-derived sleep and activity data can reliably predict relapse or guide clinical decision-making in routine psychosis care remains to be demonstrated in prospective, adequately powered trials with clinically meaningful primary endpoints.

### 6.3. Ecological Momentary Assessment and Just-in-Time Adaptive Interventions

Ecological Momentary Assessment (EMA), the repeated and intensive sampling of experiences, symptoms, and behaviors in daily life through the use of electronic devices, has yielded significant insights into the nuanced, bidirectional dynamics of the relationship between sleep and psychosis that cannot be captured through cross-sectional designs. Studies utilizing EMA within CHR populations have demonstrated that nightly sleep fragmentation anticipates increased paranoia and unreality experiences the following day. Furthermore, heightened psychotic-like experiences are associated with subsequent sleep disturbances, with effect sizes indicating clinically meaningful coupling at the day level [23]. However, the implementation of EMA in psychosis populations is not without methodological challenges. The cognitive burden associated with repeated ecological sampling—typically involving 6–10 daily prompts over periods of 6 or more days—may disproportionately affect individuals with attentional deficits, working memory impairment, or disorganized cognition, resulting in lower completion rates than those observed in general population samples. Furthermore, the hypervigilant monitoring of internal states inherent to EMA protocols may risk amplifying ruminative processes or paranoid ideation in individuals with threat-based cognitive biases, warranting careful attention to sampling frequency, burden minimization, and clinical monitoring in EMA study designs targeting this population [63].

Just-in-Time Adaptive Interventions (JITAIs)—digitally delivered, personalized interventions that respond to real-time data from wearable sensors and EMA—offer a promising framework for delivering targeted sleep support precisely when and where it is most needed. Nahum-Shani and colleagues have articulated the theoretical and design foundations for JITAIs in mental health, formalizing key components—including tailoring variables, decision rules, and intervention options—and identifying schizophrenia as a high-priority application domain [63]. In this context, FOCUS—a smartphone-based behavioral intervention for schizophrenia—represents a pioneering implementation of JITAI principles in psychosis, prompting users three times daily to assess their status across five clinical domains, including sleep, mood regulation, and coping with hallucinations, and delivering tailored self-management strategies when difficulties are detected [64,65,66,67].

### 6.4. Artificial Intelligence and Precision Sleep Medicine in Psychosis

AI and machine learning methods are increasingly applied to sleep data in psychiatry, spanning phenotyping, outcome prediction, and treatment optimization. Skeldon et al. demonstrated that a validated mathematical model of the two-process regulation of sleep, fitted to individual actigraphy and melatonin data from patients with schizophrenia, could generate personalized predictions of the light-exposure interventions needed to restore normative sleep–wake timing, translating complex neuroscience into individually tailored, actionable clinical recommendations [68]. More broadly, AI-driven analysis of passively collected digital phenotyping data, including smartphone usage patterns, GPS mobility, screen time, and accelerometer-derived physical activity, has shown the ability to predict symptom exacerbation and relapse in psychosis with meaningful lead times.

While these developments are scientifically compelling, the application of AI and machine learning to sleep data in psychosis remains largely at the proof-of-concept stage. Predictive models have been validated in small, selected cohorts under research conditions, but their generalizability to routine clinical settings, heterogeneous patient populations, and real-world implementation contexts remains unestablished. Interpretability, data governance, equitable access, and integration with existing electronic health record systems remain unresolved translational challenges that must be addressed before AI-driven precision sleep medicine can be responsibly recommended for clinical adoption.

## 7. Barriers to Implementation and Clinical Recommendations

### 7.1. Barriers at the Clinician and Service Level

Multiple interacting barriers contribute to the persistent undertreatment of sleep disorders in individuals with psychosis. At the clinician level, inadequate training in sleep medicine and cognitive behavioral therapy for insomnia (CBT-I) during psychiatric residency constitutes a fundamental obstacle—the majority of psychiatrists and mental health professionals lack the necessary knowledge and skills to administer CBT-I or adapt it for patients with psychosis.

Moreover, postgraduate educational opportunities specifically tailored to the intersection of sleep medicine and psychiatric practice remain scarce; dedicated courses, advanced training programs, and master-level curricula addressing sleep disturbances in the context of psychosis are largely absent from the continuing medical education landscape. Therapeutic nihilism—the implicit belief that sleep disturbances in psychosis are simply consequences of the primary illness or its medication—remains widespread in clinical culture despite evidence to the contrary. Attributional barriers, where sleep problems are reflexively attributed to antipsychotic sedation rather than recognized as independently treatable conditions, further diminish the likelihood of implementing targeted sleep interventions.

At the service level, limited access to clinical psychologists and therapists trained in CBT-I creates a bottleneck that prevents even motivated clinicians from referring patients for appropriate treatment. Waiting times for psychological therapies in most public mental health systems are long, and sleep-focused psychological interventions are rarely commissioned as stand-alone services within psychosis care pathways. Time constraints during psychiatric outpatient appointments—typically 20–30 min—further hinder comprehensive sleep assessment and brief psychoeducational interventions.

### 7.2. Barriers at the Systemic Level

From a systemic perspective, the lack of recognition of sleep medicine as an integral component within psychiatric training curricula and ongoing professional development initiatives constitutes a structural deficiency that sustains skill gaps among successive cohorts of mental health practitioners. Historically, national and regional clinical guidelines for psychosis management, including NICE and the Schizophrenia Patient Outcomes Research Team (PORT) guidelines, have given limited emphasis to sleep assessment and management, thereby diminishing its clinical significance relative to positive symptoms and medication adherence. Funding streams allocated to mental health services seldom encompass provisions specific to sleep, and the integration of sleep medicine specialists within multidisciplinary mental health teams remains virtually nonexistent. Additionally, the absence of diagnostic coding and reimbursement pathways for sleep disorders in psychiatric settings, where clinicians predominantly focus on ICD or DSM-based primary psychiatric diagnoses, further hampers the systematic identification and documentation of comorbid sleep conditions. Overcoming these systemic barriers necessitates coordinated efforts involving revisions to postgraduate training curricula, clinical guideline updates, service commissioning frameworks, and health informatics systems.

### 7.3. Barriers at the Patient Level

Barriers at the patient level to engagement with sleep interventions in psychosis are considerable. Cognitive impairments, including deficits in attention, working memory, processing speed, and executive function, may hinder the acquisition and application of CBT-I techniques, particularly in the completion of sleep diaries and cognitive restructuring. Negative symptoms, such as amotivation and avolition, might diminish the willingness to invest effort in behavioral interventions that require consistent daily adherence. Moreover, stigma and the normalization of poor sleep, where many patients perceive disrupted sleep as an inevitable aspect of their condition, further discourage help-seeking behavior. Additionally, assumptions cannot be made regarding digital literacy, access to smartphones and reliable internet connections, and comfort with technology-mediated health interventions across the socioeconomically diverse populations served by psychosis services. This creates a risk of digital exclusion, which must be addressed proactively in the design and implementation of digital sleep solutions.

### 7.4. Evidence-Informed Clinical Recommendations

Based on the reviewed evidence, we propose the following clinical recommendations informed by current research. First, sleep assessments should be routinely conducted at each clinical encounter in psychosis settings, utilizing brief, validated instruments such as the Insomnia Severity Index (ISI) or PSQI. These assessments should encompass insomnia, hypersomnia, nightmare disorder, and symptoms indicative of obstructive sleep apnea (OSA), with explicit inquiries regarding suicidal ideation in patients exhibiting significant sleep complaints.

Second, findings from sleep assessments should be documented within the clinical record and utilized to inform risk assessments, recognizing insomnia as a modifiable and independent risk factor for suicide, thereby necessitating targeted sleep interventions and appropriate safety planning.

Third, clinicians should be equipped, through adequate training and supervision, to administer brief psychoeducation on sleep, basic stimulus control strategies, and sleep hygiene advice as initial responses to insomnia complaints, with established referral pathways for more comprehensive CBT-I.

Fourth, digital CBT-I tools adapted specifically for individuals with psychosis, or hybrid digital–human formats with minimal therapist guidance, should be considered accessible and scalable second-line options, particularly in resource-constrained environments, while fully automated programs should be employed cautiously until supported by dedicated RCT evidence.

Fifth, interventions targeting circadian rhythms, such as structured daily exposure to bright light, consistent sleep–wake schedules, and activity-based rhythm stabilization, should be integrated into care plans for patients exhibiting circadian dysregulation, complementing CBT-I designed for distinct sleep phenotypes.

Sixth, healthcare services should invest in training at least one specialist per team in the delivery of adapted CBT-I and related sleep interventions for individuals with psychosis, establishing a sustainable pathway for evidence-based sleep treatment within current clinical frameworks. The six evidence-informed clinical recommendations derived from the reviewed literature are summarized in Table 3. Recommendations 1–3 are considered immediately implementable within existing clinical frameworks. Recommendations 4–6 address strategies that, while clinically promising, require further implementation and efficacy research before they can be confidently adopted in routine psychosis services.

## 8. Discussion

This narrative review synthesizes a rapidly growing body of evidence showing that sleep disturbances are clinically central, mechanistically plausible, and therapeutically tractable features of the psychosis spectrum. Across the illness course, from CHR states through FEP to chronic schizophrenia, sleep disruption is near-universal, multifaceted in its phenomenology, and bidirectionally linked to psychotic, affective, and cognitive symptoms via identifiable neurobiological and psychological mechanisms.

A critical comparison of available interventions reveals complementary strengths and indications. CBT-I, whether delivered individually, in groups, or digitally within a stepped-care framework, most directly addresses the subjective complaint of insomnia and has the strongest evidence base. However, its efficacy in patients with primary circadian dysfunction, phase delays, non-24 h cycles, or highly irregular sleep–wake patterns is limited because these disorders involve a mismatch in sleep timing rather than impaired sleep initiation or maintenance. For these patients, circadian-focused approaches such as L-DART or light therapy are more directly indicated and may need to precede or accompany CBT-I. IRT specifically addresses nightmare disorder and should be considered for any patient for whom nightmares are a prominent complaint. Digital approaches offer critical advantages in accessibility and scalability but require psychosis-specific adaptation and robust evaluation before they can be confidently recommended as alternatives to face-to-face treatment. The stepped-care framework provides a rational and resource-efficient structure for integrating these complementary approaches within existing clinical pathways.

A note of caution is warranted regarding digital and AI-driven approaches. Although these technologies are widely discussed for their theoretical promise and accessibility advantages, the evidence for their clinical effectiveness in psychosis is currently insufficient to support confident recommendations. Most studies are single-arm feasibility investigations with no control condition, small samples, and short follow-up; long-term outcomes, safety in populations with active psychotic symptoms, and scalability under routine service conditions have not been systematically evaluated. These approaches should therefore be positioned as emerging research priorities rather than established components of clinical care.

## 9. Limitations of the Evidence Base and Future Research Directions

Several key gaps in the current evidence base shape the research agenda for the field. First, adequately powered, well-controlled RCTs of adapted CBT-I and other non-pharmacological sleep interventions in psychosis populations, including both early and chronic illness stages, are urgently needed. Sample sizes in existing trials have been too small to detect moderate effect sizes with adequate power, and primary outcome measures have varied widely, impeding cumulative synthesis. Pre-registering trials and adopting standardized outcome measures, including validated self-report instruments (ISI, PSQI) and objective sleep parameters (actigraphy), would substantially improve comparability and interpretability.

Second, the field requires longitudinal studies with repeated, standardized sleep assessments to characterize the temporal dynamics of the sleep–psychosis relationship, particularly the directionality of associations between sleep and symptom changes over time. EMA designs embedded within clinical trials offer a particularly powerful approach, enabling simultaneous testing of mechanistic hypotheses and identification of individual-level predictors of treatment response. Third, the neurobiological mechanisms mediating the effects of behavioral sleep interventions on psychotic symptoms—including changes in sleep spindle density, dopaminergic function, circadian phase, and affective regulation—have not been systematically studied in psychosis intervention trials and represent important targets for translational research that integrates neuroimaging and electrophysiology with clinical outcomes.

Fourth, developing and evaluating psychosis-specific digital sleep interventions, incorporating cognitive accommodations, safety monitoring, and hybrid human-digital delivery formats, is a research priority, given the accessibility advantages of digital approaches and the current lack of evidence for their efficacy in clinical psychosis populations. Fifth, the potential of sleep-focused interventions to prevent psychosis onset in CHR populations, reduce relapse rates in established psychosis, and reduce suicidal risk in FEP, all highly clinically significant outcomes, has not been evaluated in adequately powered prospective trials and should be prioritized in future research funding and commissioning. Sixth, future work should explicitly address equity dimensions, ensuring that trial populations are representative of the socioeconomically, culturally, and ethnically diverse communities served by psychosis services and that digital interventions are designed with equitable access in mind.

Seventh, the neurobiological mechanisms discussed in this review are derived predominantly from studies of schizophrenia and schizophrenia-spectrum disorders. Affective psychoses, bipolar disorder with psychotic features, and other psychotic conditions are underrepresented in the mechanistic literature on sleep and psychosis, and it cannot be assumed that the described pathophysiological pathways apply uniformly across diagnostic categories.

A further limitation of the current review is its insufficient attention to clinical heterogeneity within psychosis populations. Factors such as illness stage, antipsychotic exposure, comorbid depressive and anxiety symptoms, trauma history, PTSD, substance use, metabolic syndrome, obesity, cognitive impairment, negative symptoms, social isolation, and comorbid sleep disorders are likely to substantially influence both the phenomenology of sleep disturbances and the acceptability and effectiveness of sleep-focused interventions. Future research should systematically examine how these clinical variables moderate treatment response across CHR, FEP, and chronic schizophrenia populations, and intervention protocols should be designed with sufficient flexibility to accommodate this heterogeneity.

Future research should explicitly address sleep neurophysiology and the effects of sleep-focused interventions across the full diagnostic breadth implied by the term ‘psychosis spectrum.’

Finally, the optimal coordination of pharmacological and behavioral sleep treatment in psychosis, including the impact of specific antipsychotic compounds on sleep architecture and the timing of CBT-I relative to medication optimization, warrants systematic investigation to guide integrated treatment planning.

Several limitations of the review should be acknowledged to ensure transparency and consistency with SANRA principles. First, although the search strategy was structured, it was not exhaustive in the strict sense of a systematic review, and selection bias in source identification cannot be ruled out. Second, the search was restricted to English-language publications, potentially omitting relevant findings from non-English literature. Third, the included studies are highly heterogeneous in design, population characteristics, illness stage, outcome measures, and follow-up duration, limiting comparability across sections. Fourth, no formal risk-of-bias assessment was conducted for individual studies, and evidence quality was appraised narratively rather than through validated grading tools such as GRADE. Fifth, as with any narrative synthesis, the thematic integration of findings is subject to interpretive choices that may not be fully reproducible. These limitations should be considered when interpreting the conclusions and recommendations presented in this review.

## 10. Conclusions

Sleep disturbances are a clinically important, mechanistically plausible, and therapeutically tractable target for intervention across the full psychosis spectrum. The evidence reviewed in this paper supports a reconceptualization of sleep in psychosis: from an epiphenomenal accompaniment of psychiatric illness to a mechanistically plausible, transdiagnostically significant, and potentially modifiable determinant of clinical outcome—a hypothesis that warrants confirmation in adequately powered longitudinal and interventional studies.

Non-pharmacological interventions, led by CBT-I within a stepped-care framework and complemented by circadian-focused approaches, IRT, and mindfulness-based methods, offer an expanding therapeutic toolkit with a growing evidence base. Digital health technologies, including smartphone applications, wearable sensors, EMA platforms, and AI-driven personalization, hold substantial promise for extending the reach and accessibility of evidence-based sleep care to populations for whom specialist psychological services have historically been limited. Engaging caregivers and family members as active partners in sleep-focused intervention represents an underutilized resource with the potential to improve both treatment adherence and caregiver well-being.

The field now faces a clear translational imperative. Several specific actions are needed with urgency. Sleep outcomes, including both self-reported insomnia severity and objective actigraphic parameters, should be adopted as pre-specified secondary endpoints in all future early intervention in psychosis trials, RCTs of antipsychotic medications, and suicide prevention studies in this population; their current near-universal absence from such trials represents a missed opportunity to generate actionable clinical data at scale. National and international clinical guidelines for psychosis should be updated to include explicit recommendations for routine sleep assessment and evidence-based sleep treatment, providing the normative clinical authority that individual clinicians and services require to prioritize sleep in resource-constrained environments. Finally, collaborative networks between sleep researchers, early intervention clinicians, digital health developers, people with lived experience of psychosis, and caregivers should be established to co-design the next generation of sleep interventions, ensuring that they are not only effective but genuinely acceptable, accessible, and equitable for the diverse communities they aim to serve.

Sleep, long relegated to the margins of psychosis clinical care, warrants a more central position in both clinical practice and research. While the evidence base for non-pharmacological sleep interventions in psychosis is still maturing, the convergence of epidemiological, neurobiological, and early interventional data is sufficient to justify systematic investment in routine sleep assessment and the implementation of evidence-based sleep treatment within psychosis services.

## Figures and Tables

**Table 1:** Pattern of sleep disturbances across stages of the psychosis spectrum.

Sleep Disturbance	CHR	FEP	Chronic Psychosis
Insomnia disorder	++	+++	+++
Nightmare disorder	+	++	+++
Hypersomnia	+	++	+++ *
Obstructive sleep apnea	n.a.	+	+++
Circadian rhythm disorders	+++	++	+

+++ well-documented; ++ reported, limited data; + sparse/anecdotal evidence; n.a. no data available. * partly confounded by antipsychotic medication.

**Table 2:** Summary of key non-pharmacological and digital sleep intervention studies in psychosis.

Study (Year)	Population	Intervention	Design	N	Sleep Outcome	Psychosis/Clinical Outcome	Follow-Up
Freeman et al. (2020) [8]	Schizophrenia spectrum, persecutory delusions	Face-to-face adapted CBT-I	Pilot RCT	50	Significant reduction in ISI	Reduction in persecutory delusions and depression	3 months
Sheaves et al. (2019)—CBT-I psychosis [52]	Schizophrenia, persecutory delusions	Face-to-face adapted CBT-I	Pilot RCT	20	Significant reduction in insomnia (ISI)	Trend toward reduction in delusion severity and paranoia	3 months
Taylor et al. (2022) [57]	CHR-P, insomnia	Adapted CBT-I case series	Case series	5	Improvements in sleep onset latency and PSQI scores	Reductions in attenuated psychotic symptoms and anxiety	End of therapy
Waite et al. (2023)—SleepWell CHR-P [47]	Ultra-high risk (CHR-P), insomnia	12-week tailored psychological sleep therapy	Feasibility RCT	40	Very large ISI reduction (d = 2.67); sustained at 9m (d = 1.91)	Reductions in paranoia, anxiety, depression at 9-month follow-up; no transitions to psychosis	9 months
Faulkner et al. (2023)—L-DART [21]	Schizophrenia spectrum, diverse sleep phenotypes	12-week Light-Dark & Activity Rhythm Therapy (OT-delivered)	Single-arm mixed-methods	10	Improvements in subjective sleep quality and circadian timing	Improvements in wellbeing, occupational function, and daily activity rhythms	End of therapy
Tsai et al. (2024) [48]	Schizophrenia inpatients	Circadian rhythm simulation lighting (CRSL) system	Prospective cohort	20	Improved sleep timing; reduced nocturnal awakening	Improvement in BPRS total score; improved cognitive performance (MMSE)	10 weeks
Beattie et al. (2023) [44]	Psychosis spectrum	Smartphone-based guided CBT for sleep (hybrid)	Single-arm feasibility	28	Significant ISI reduction; improved subjective sleep quality	Improvements in depression and quality of life; high user satisfaction	6 weeks
Jeon et al. (2024)—Sleep Catcher [58]	Schizophrenia, cognitive impairment	CBT-I app with wearable-automated sleep diary	Beta feasibility	5	Automated sleep diary feasible; objective TST accurately tracked	Not assessed (feasibility phase)	2 weeks
Tanskanen et al. (2024)—ongoing [45]	Schizophrenia, schizoaffective	Internet CBT-I vs. group CBT-I vs. TAU	Multi-arm RCT (ongoing)	84–120 (target)	ISI (primary outcome)	Psychotic symptoms, QoL, social functioning	12 months

BPRS = Brief Psychiatric Rating Scale; CBT-I = Cognitive Behavioral Therapy for Insomnia; CHR-P = clinical high-risk for psychosis; ISI = Insomnia Severity Index; L-DART = Light-Dark and Activity Rhythm Therapy; MMSE = Mini-Mental State Examination; OT = occupational therapist; PSQI = Pittsburgh Sleep Quality Index; QoL = quality of life; RCT = randomized controlled trial; TAU = treatment as usual; TST = total sleep time.

**Table 3:** Evidence-informed clinical recommendations for sleep disturbances in psychosis.

#	Recommendation	Content	Target	Key Tools
**1**	**Routine sleep assessment**	Sleep screening should be conducted at every clinical encounter using brief validated instruments. Assessment should encompass insomnia, hypersomnia, nightmare disorder, and OSA symptoms; suicidal ideation must be explicitly explored in patients with significant sleep complaints.	All patients	ISI, PSQI
**2**	**Documentation and risk assessment**	Sleep assessment findings should be recorded in the clinical record and integrated into risk assessments. Insomnia should be recognized as a clinically significant and potentially independent risk factor for suicidal ideation and behavior; however, this association should be interpreted in the context of frequent comorbidities, including depressive symptoms, anxiety, and severity of positive symptoms, which may partially account for or amplify the observed relationship. Risk assessment should therefore adopt a multivariate approach considering sleep disturbance alongside these co-occurring factors while fully automated programs should be employed cautiously pending dedicated RCT evidence. Wearable-informed relapse prediction, JITAIs, and AI-supported personalization should be regarded as emerging strategies requiring further implementation research and are not yet suitable for routine clinical adoption.	Clinicians	Clinical records
**3**	**Psychoeducation and first-line intervention**	Clinicians should be trained to deliver brief sleep psychoeducation, basic stimulus control strategies, and sleep hygiene advice as initial responses to insomnia complaints, with established referral pathways to more comprehensive CBT-I.	Clinicians	CBT-I (step 1)
**4**	**Digital CBT-I as a second-line option**	Digital CBT-I tools adapted for individuals with psychosis, or hybrid digital–human formats with minimal therapist guidance, should be considered as accessible and scalable second-line options; fully automated programs should be employed cautiously pending dedicated RCT evidence. Wearable-informed relapse prediction, JITAIs, and AI-supported personalization should be considered emerging research priorities rather than ready-to-implement clinical tools.	Resource-limited settings	dCBT-I, JITAIs, apps
**5**	**Circadian-focused interventions**	Structured daily bright-light exposure, consistent sleep–wake schedules, and activity-based rhythm stabilization should be integrated into care plans for patients with circadian dysregulation, complementing CBT-I tailored to distinct sleep phenotypes.	Circadian dysregulation	L-DART, light therapy
**6**	**Specialist training within clinical teams**	Healthcare services should invest in training at least one specialist per team in the delivery of adapted CBT-I and related sleep interventions for individuals with psychosis, establishing a sustainable pathway for evidence-based sleep treatment within existing clinical frameworks.	Services/teams	Training, supervision

CBT-I = Cognitive Behavioral Therapy for Insomnia; dCBT-I = digital CBT-I; ISI = Insomnia Severity Index; JITAI = Just-in-Time Adaptive Intervention; L-DART = Light-Dark and Activity Rhythm Therapy; OSA = obstructive sleep apnea; PSQI = Pittsburgh Sleep Quality Index; RCT = randomized controlled trial.

## Data Availability

No new data were created or analyzed in this study.
